# Neonatal Neuroblastoma with Inferior Vena Cava Syndrome

**Published:** 2013-05-01

**Authors:** Serdar Alan, Ufuk Cakir, Dilek Kahvecioglu, Zulfikar Gordu, Omer Erdeve, Handan Dincaslan, Begum Atasay, Serdar Beken, Gulsan Yavuz, Saadet Arsan

**Affiliations:** Division of Neonatology, Department of Pediatrics, Ankara University School of Medicine, Ankara, TURKEY; Division of Neonatology, Department of Pediatrics, Ankara University School of Medicine, Ankara, TURKEY; Division of Neonatology, Department of Pediatrics, Ankara University School of Medicine, Ankara, TURKEY; Division of Pediatric Oncology, Department of Pediatrics, Ankara University School of Medicine, Ankara, TURKEY; Division of Neonatology, Department of Pediatrics, Ankara University School of Medicine, Ankara, TURKEY; Division of Pediatric Oncology, Department of Pediatrics, Ankara University School of Medicine, Ankara, TURKEY; Division of Neonatology, Department of Pediatrics, Ankara University School of Medicine, Ankara, TURKEY; Division of Neonatology, Dr. Sami Ulus Children Hospital, Ankara, TURKEY; Division of Pediatric Oncology, Department of Pediatrics, Ankara University School of Medicine, Ankara, TURKEY; Division of Neonatology, Department of Pediatrics, Ankara University School of Medicine, Ankara, TURKEY

**Keywords:** Neuroblastoma, Inferior vena cava syndrome, Newborn

## Abstract

Neuroblastoma (NBL) is a neuroectodermal tumor derived from neural crest cells. The biological and clinical behavior of NB is extremely heterogenous. We here report a newborn who presented as 4S NBL with a massive hepatomegaly resulting in IVC syndrome.

## INTRODUCTION

Neuroblastoma (NBL) is a neuroectodermal tumor which may arise from either the adrenal gland or anywhere along the sympathetic chain [1]. Prognostic factors include age at presentation, extent of disease, and biological properties, primarily amplification of the MYC-N oncogene [2]. Newborns with 4S NBL have an unfavorable prognosis compared with 3–12 month olds. [2,3]. Congenital NBLs with massive hepatic involvement are extremely rare [4-6]. 

IVC syndrome is a result of obstruction of the IVC [7]. Although IVC syndrome due to massive hepatomegaly has been reported, in few cases, but data on its management is lacking. We report a case of rapidly progressive neonatal NBL with massive hepatomegaly complicated by inferior vena cava syndrome.

## CASE REPORT

A 4050-gram male newborn was delivered vaginally to a 20-year-old mother at 413/7 gestational weeks with no dysmorphic features and discharged on the postnatal day two. On the postnatal day six, a distended abdomen was noticed and baby was admitted to another hospital for investigations. On abdominal examination firm mass was palpable. The liver was occupying the entire abdomen. The patient also had edema of the lower extremities and mild respiratory distress. 

Initial laboratory findings including complete blood count (hemoglobin: 14.6 g/dl, white blood count: 22,800 /l, platelet count: 330,000/l), electrolytes and renal function tests were within normal range. Alanine aminotransferase level (19 U/l, N: <45 U/l) was normal. However aspartate aminotransferase (85 U/l, N: <41 U/l), gamma glutamyl transferase (238 U/l, N: 8-61 U/l) and total/direct bilirubin levels (3/1.6 mg/dl, N: <1.1/<0.3 mg/dl) were high. Serological evaluation for toxoplasma gondii, rubella, cytomegalovirus, herpes simplex, Epstein–Barr virus and parvovirus were all negative. Abdominal ultrasonography (USG) revealed hepatomegaly with a heterogeneous liver parenchyma and a 39x31 mm hypo-echoic solitary non-calcified mass in the right adrenal region. A computed tomography scan revealed a right adrenal soft tissue mass (4.8x3.6 cm) with no calcification and contrast uptake, and significant hepatomegaly. The spleen appeared to be in normal size but was replaced inferiorly because of the massive hepatomegaly. Serum neuron-specific enolase level was higher, 60 ng/mL (normal range: <10 ng/mL), and alpha feto protein level (22754 ng/mL, N: >45000 ng/mL at birth) was normal. Urine vanillylmandelic acid and serum ferritin levels were 5718 mg/day (N: 0.5-2 mg/day) and 531 ng/mL (N: 24-336 ng/mL) retrospectively. Bone marrow aspiration revealed no malignant infiltration, no rosette forming and no storage cells. On the postnatal day eighteen, doppler USG showed decreased flow in the IVC, secondary to compression. Based on clinical presentation and imaging modalities, the presumptive diagnosis of NBL was made and the case was referred to our neonatal intensive care unit (NICU) on the postnatal day twenty. 

On admission to our NICU, he had respiratory distress with 40% O2 requirement and significant abdominal distension with visible thin venous vessels through his abdominal skin (Fig. 1a). He had tachypnea besides edema of lower extremities, scrotum and penile area. Complete blood count revealed anemia (hemoglobin: 9.5 g/dl), leukocytosis (33.300 /l) and mild thrombocytosis (536.000/l). Serum sodium (128 mEq/l), chloride (94 mEq/l), calcium (7 mg/dl) and phosphorus (0.7 mg/dl) concentrations were low. Serum uric acid and renal function tests revealed normal results (uric acid: 5.8 mg/dl, blood urea nitrogen: 9 mg/dl, creatinine: 0.43 mg/dl). Liver function tests revealed cholestasis (alanine aminotransferase: 40 U/l, aspartate aminotransferase: 101 U/l, γ-glutamyltransferase: 463 U/l, total/direct bilirubin: 3.05/2.02 mg/dl) and hypoproteinemia (total protein: 3.4 g/dl and albumin: 1.8 g/dl). Clotting studies demonstrated prolonged prothrombin time (PT) as 16 seconds (N: 9.4 to 12.5 seconds) and low fibrinogen level (0.89 g/dl, N: 2.38–4.98). 

Second abdominal USG evaluation showed right suprarenal mass which was about 5 cm in diameter, severely diffuse hepatomegaly and ascites. After pediatric oncology consultation, the patient underwent hydration, chemotherapy and allopurinol treatments between the postnatal days 20–23. The patient received modified VEC (vincristine, etoposide, carboplatin) protocol due to poor clinical condition. In spite of the received chemotherapy, his clinical condition worsened progressively. The abdominal distention was so increased that he required high frequency oscillatory ventilation support. However, he did not develop signs of tumor lysis syndrome. The patient was accepted as chemotherapy-resistant NBL due to clinical deterioration with massive liver involvement on the 3rd day of treatment and surgical excision was performed (Fig. 1b, 1c). Pathology revealed intra-adrenal tumor with wide-spread expression of bcl 2 in addition to distant metastases in the liver and minimal lymphocytic infiltration. MYC-N amplification obtained from adrenal specimen revealed <3 copies. IVC compression directly observed during the surgery resulted in severe circulation collapse and the patient died due to hypotensive shock and disseminated intravascular coagulation just two hours after the operation.

**Figure:**
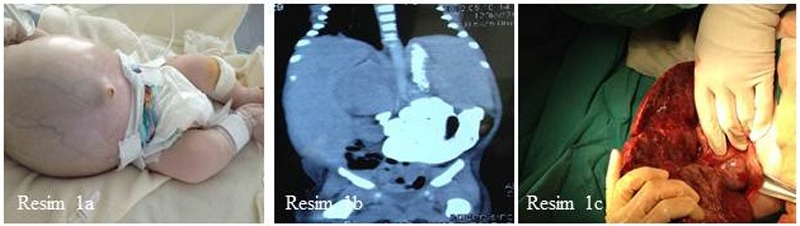
Figure 1: a) Significant abdominal distension with thin venous vessels on abdominal skin was noticed b) computed tomography scan revealed massive hepatomegaly and right adrenal tumor mass c) tumor mass just close to liver and massive hepatomegaly view in operation.

## DISCUSSION

Stage 4S NBL is unique among metastatic cancers in that it may regress without treatment, and is thus associated with a favorable outcome [8, 9]. Although, stage 4S NBLs tends to undergo spontaneous regression and NBLs in infants often remit with minimal therapy, stage 4S can result in higher morbidity and mortality [5, 8]. Furthermore, few neonates with NBL associated with massive hepatic metastases similar to our presented case were reported to have poor clinical course. There are multifactorial reasons for poor clinical outcome like massive hepatic tumor burden leading to hypoalbuminemia, edema, and coagulopathy with respiratory failure due to a combination of a small thoracic cavity secondary to massive hepatomegaly and pulmonary hemorrhage precipitated by abnormal coagulation [5]. The hepatomegaly can also cause mechanical compression of vascular structures and IVC syndrome which can become life threatening immediately [8]. 

Inferior vena cava syndrome can be caused by thrombosis besides mechanical compression. The prevalence of tumor thrombus in the IVC for Wilms tumors has been reported to be as high as 19% [10]. Although vascular invasion by neuroblastoma is rare, intracaval tumor thrombus have been described in several isolated case reports. Six such cases have been described in children, including 1 with right atrial extension of the tumor thrombus [11, 12]. Doppler USG and intraoperative examination did not suggest thrombi to be the cause of IVC syndrome in our patient. We suggest that mechanical compression was the only factor that played a role in the etiology. 

MYC-N amplification is recognized as a strong marker for poor prognosis in NBL at all ages [4]. In our patient there was no MYC-N amplification. In the absence of life-threatening symptoms, a ‘‘wait and see’’ strategy is adopted neonatal NBL cases. Boztug et al, reported successful treatment of a progressive stage 4S neuroblastoma in a neonate with hepatic artery embolization in addition to multimodality treatment [2]. However, rapidly progressive deterioration and chemotherapy resistance in our patient led us to surgical intervention.

In conclusion, there is still lack of knowledge on approach to these patients. We suggest that massive hepatomegaly can deteriorate the patient’s clinical condition and they may require either embolization or surgery as an urgent management.

## Footnotes

**Source of Support:** Nil

**Conflict of Interest:** None declared
